# A Rapidly Evolving Polybasic Motif Modulates Bacterial Detection by Guanylate Binding Proteins

**DOI:** 10.1128/mBio.00340-20

**Published:** 2020-05-19

**Authors:** Kristin M. Kohler, Miriam Kutsch, Anthony S. Piro, Graham D. Wallace, Jörn Coers, Matthew F. Barber

**Affiliations:** a Institute of Ecology & Evolution, University of Oregon, Eugene, Oregon, USA; b Department of Molecular Genetics and Microbiology, Duke University Medical Center, Durham, North Carolina, USA; c Department of Immunology, Duke University Medical Center, Durham, North Carolina, USA; d Department of Biology, University of Oregon, Eugene, Oregon, USA; University of Pittsburgh

**Keywords:** *Shigella*, cell-autonomous immunity, evolution, guanylate binding proteins, host-pathogen interactions

## Abstract

Cell-autonomous immunity relies on the rapid detection of invasive pathogens by host proteins. Guanylate binding proteins (GBPs) have emerged as key mediators of vertebrate immune defense through their ability to recognize a diverse array of intracellular pathogens and pathogen-containing cellular compartments. Human and mouse GBPs have been shown to target distinct groups of microbes, although the molecular determinants of pathogen specificity remain unclear. We show that rapid diversification of a C-terminal polybasic motif (PBM) in primate GBPs controls recognition of the model cytosolic bacterial pathogen Shigella flexneri. By swapping this membrane-binding motif between primate GBP orthologs, we found that the ability to target S. flexneri has been enhanced and lost in specific lineages of New World primates. Single substitutions in rapidly evolving sites of the GBP1 PBM are sufficient to abolish or restore bacterial detection abilities, illustrating a role for epistasis in the evolution of pathogen recognition. We further demonstrate that the squirrel monkey GBP2 C-terminal domain recently gained the ability to target S. flexneri through a stepwise process of convergent evolution. These findings reveal a mechanism by which accelerated evolution of a PBM shifts GBP target specificity and aid in resolving the molecular basis of GBP function in cell-autonomous immune defense.

## Abstract (precis)

Many infectious diseases are caused by microbes that enter and survive within host cells. Guanylate binding proteins (GBPs) are a group of immune proteins which recognize and inhibit a variety of intracellular pathogenic microbes. We discovered that a short sequence within GBPs required for the detection of bacteria, the polybasic motif (PBM), has been rapidly evolving between primate species. By swapping PBMs between primate GBP1 genes, we were able to show that specific sequences can both reduce and improve the ability of GBP1 to target intracellular bacteria. We also show that the ability to envelop bacteria has independently evolved in GBP2 of South American monkeys. Taking the results together, this report illustrates how primate GBPs have adapted to defend against infectious pathogens.

## INTRODUCTION

Diverse metazoan cell types possess the innate ability to resist infection by pathogens, a feature termed cell-autonomous immunity. Detection of intracellular bacteria, viruses, or eukaryotic parasites by host factors engenders cell-autonomous defense programs operating to contain or eliminate invasive pathogens from an infected cell (1). These defense programs can be activated in response to proinflammatory interferons produced by professional immune cells or neighboring infected cells. Interferon signaling prompts the expression of interferon-stimulated genes (ISGs) which encode a wide range of antimicrobial proteins (2). Among the most highly upregulated ISGs are the members of a class of dynamin-related cytoplasmic GTPases called guanylate binding proteins, or GBPs. Vertebrate GBPs contribute to defense against diverse pathogens, and GBP function has also been implicated in the regulation of inflammation (3–5).

GBPs consist of an N-terminal catalytic GTPase domain followed by an elongated helical domain which mediates interactions with target proteins or membranes. GTP binding and hydrolysis promote the dimerization, oligomerization, and polymerization of GBPs as well as recruitment of additional GBP family members (6). Oligomerization of GBPs on pathogen-containing membrane-bound compartments prompts an array of antimicrobial activities, including the production of radical oxygen species by corecruited oxidases, the fusion of these compartments with degradative lysosomes, their encapsulation within autophagosome-like structures, and the lytic disintegration of microbe-containing compartments (7). Some GBPs also possess the ability to target microbes that reside inside the host cell cytosol. Cytosolic bacteria enclosed by GBPs undergo lytic destruction in mouse macrophages (8–10) or are blocked from engaging the host actin polymerization machinery in human epithelial cells, thereby losing the ability to disseminate (11, 12). The importance of GBPs as potent immune effectors is further illustrated by the recent discovery that the enteric bacterial pathogen Shigella flexneri injects host cells with the virulence factor IpaH9.8, which specifically disrupts GBP function (11–13). IpaH9.8 is an E3 ubiquitin ligase that directly binds to several GBP family members, targeting them for destruction by the proteasome (12–14). While IpaH9.8 is the only microbial GBP antagonist reported so far, it is likely that additional pathogen-encoded GBP countermeasures remain to be discovered.

Despite a wealth of evidence supporting the idea of a role of GBPs in cell-autonomous host defense, the molecular mechanisms underlying GBP function and target specificity remain enigmatic. One informative observation is that mammalian GBPs target cytosolic microbes as well as microbe-associated membranous structures in a hierarchical manner, with individual GBPs functioning as “pioneers” that recruit other family members through heterotypic interactions (15, 16). In particular, GBP1, GBP2, and GBP5 in humans are predicted to directly associate with target membranes due to the presence of a C-terminal CaaX box leading to posttranslational prenylation, which acts as a hydrophobic lipid anchor (6). In support of this model, it was previously shown that recombinant human GBP1 (hGBP1) binds directly to lipid bilayers *in vitro* in a GTP- and prenylation-dependent manner (17). However, prenylation alone is unlikely to provide targeting specificity and other protein motifs are expected to enable prenylated hGBPs to discriminate between “self” and “nonself” membranes inside infected cells (18). Consistent with this hypothesis, we previously demonstrated that hGBP1 is unique among all human GBPs in its ability to target cytosolic S. flexneri due to the presence of a polybasic motif (PBM) positioned immediately adjacent to its C-terminal CaaX box (11). While the GBP1 PBM appears critical for recognition of S. flexneri in the host cytosol, the underlying molecular mechanism remains unknown.

The unique ability of hGBP1 among all human GBPs to target cytosolic Gram-negative bacteria, the expansion of the *GBP* gene family in humans and other species, and the diversity of targets recognized by distinct GBP isoforms suggest a model in which individual GBPs have evolved unique characteristics to recognize and respond to pathogens spanning the entire tree of life. It is also notable that mouse Gbp2, the closest murine homolog of hGBP1, lacks a clearly defined C-terminal PBM and yet is capable of recognizing and eliminating cytosolic S. flexneri (13), indicating some variability in the molecular interactions that promote bacterial detection by GBPs. Collectively, these findings suggest that the divergence of GBPs within and between host genomes has drastically shifted bacterial recognition function, potentially in response to antagonistic coevolution with pathogens. In the current study, we set out to address this issue, focusing on a subset of primate GBPs which possess the ability to specifically recognize and bind cytosolic bacteria. While genetic variation in GBPs is likely to alter recognition of various microbes, we focused our investigation on S. flexneri as a model cytosolic Gram-negative bacterium whose virulence is strongly diminished by GBP recruitment. Moreover, we conjecture that variation in cytosolic bacterial surfaces might have provided a potent selective force for GBP adaptation across vertebrates. Through a combination of phylogenetic and experimental approaches, we found that accelerated evolution of membrane-targeting motifs in GBP1 and GBP2 has led to repeated gain, loss, and enhancement of bacterial detection abilities in primates.

## RESULTS

### Divergence and evolution of prenylated GBPs in simian primates.

**FIG 1:**
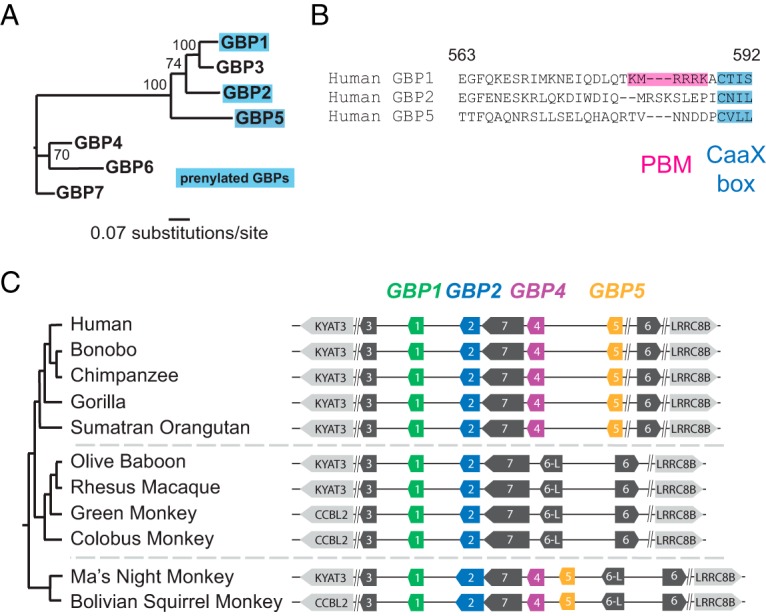
Diversity and evolution of primate guanylate binding proteins. (A) Maximum-likelihood phylogeny of the seven human GBP family members. GBPs which are known to undergo posttranslational prenylation are highlighted. Bootstrap values at nodes are based on results from 1,000 replicates. (B) Amino acid alignment of the C-terminal regions of human GBP1, GBP2, and GBP5. The location of the CaaX motif, which undergoes prenylation, and the location of the polybasic motif (PBM) of GBP1 are highlighted. Amino acid numbering is relative to human GBP1. (C) Diagram of the GBP gene cluster from representative primate genomes, illustrating the apparent loss of GBP4 and GBP5 in Old World monkeys.

We chose to focus our initial investigation on the prenylated primate GBPs (GBP1, GBP2, and GBP5; Fig. 1A) which are predicted to directly interact with intracellular microbes or microbe-derived membranous structures such as bacterial outer membrane vesicles (19, 20). While human GBP1, GBP2, and GBP5 all possess the CaaX motif required for posttranslational prenylation, GBP1 alone possesses a PBM which contributes to cytoplasmic bacterial recognition (Fig. 1B). We first noted a large-scale genomic deletion encompassing the *GBP5* locus in several Old World monkeys, suggesting that GBP5 is absent in this family (Fig. 1C). Alignment of GBP1 and GBP2 amino acid sequences from simian primates resulted in another surprising observation. While the C-terminal CaaX box is highly conserved among GBP1 and GBP2 orthologs, the amino acid sequence immediately adjacent exhibits an extreme degree of amino acid divergence (see Fig. S1 and S2 in the supplemental material). Notably, this region encompasses the C-terminal PBM of hGBP1, a protein motif essential for the hGBP1-mediated recognition of cytosolic S. flexneri in human epithelial cells (11). We considered why a domain that is required for pathogen recognition might be subject to such extreme sequence variation, despite strict conservation of the CaaX box. One possible explanation for this divergence is that prenylation of the GBP1 and GBP2 CaaX box confers general membrane-anchoring properties, whereas the adjacent C-terminal amino acid sequences allow these GBPs to discriminate between microbial nonself and self membrane surfaces. Rapid diversification of GBP1 and GBP2 orthologs in this case suggests the existence of repeated evolutionary conflicts between cytoplasmic pathogens and GBPs, in which pathogen-mediated alterations to membrane surface molecules enable evasion of GBP targeting. Our initial observations revealing elevated genetic diversity in the C-terminal regions of primate GBP1 and GBP2 thus mandated further evolutionary and experimental investigation.

10.1128/mBio.00340-20.1 FIG S1 Amino acid alignments of GBP1 orthologs surrounding sites of elevated *dN*/*dS* ratios as identified by the use of PAML and HyPhy software. Amino acid numbers are relative to human GBP1. Download FIG S1, PDF file, 0.3 MB. Copyright © 2020 Kohler et al. 2020 Kohler et al. This content is distributed under the terms of the Creative Commons Attribution 4.0 International license.

10.1128/mBio.00340-20.2 FIG S2 Amino acid alignments of GBP2 orthologs surrounding sites of elevated *dN*/*dS* ratios as identified by PAML and HyPhy. Amino acid numbers are relative to human GBP2. Download FIG S2, PDF file, 0.3 MB. Copyright © 2020 Kohler et al. 2020 Kohler et al. This content is distributed under the terms of the Creative Commons Attribution 4.0 International license.

### Rapid diversification of primate GBP1 and GBP2 C-terminal domains.

**FIG 2:**
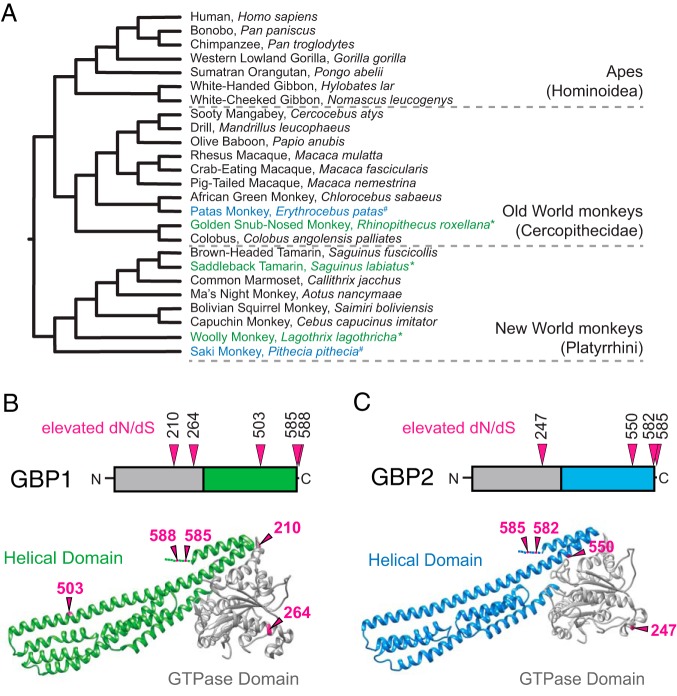
Accelerated evolution of the C-terminal polybasic motif of primate GBP1 and GBP2. (A) Species tree of simian primates used for phylogenetic analyses. Species in black were included in both the GBP1 and GBP2 data sets. Asterisks (*) indicate species that were included in GBP1 analysis alone. Crosshatch symbols (#) indicate species that were included in GBP2 analysis alone. (B) Sites in GBP1 (PDB ID 1DG3) that exhibited a statistically significant elevation in *dN*/*dS* ratio, indicative of repeated positive selection. Site numbers correspond to human GBP1 and were identified using at least four separate inference methods in the PAML and HyPhy software packages. (C) Sites in GBP2 that displayed a statistically significant elevation in *dN*/*dS* ratio, indicative of repeated positive selection. Site numbers correspond to human GBP2 and were determined using at least four separate inference methods as described for panel B. A three-dimensional predicted structure of human GBP2 was generated using the I-Tasser modeling program based on human GBP1.

If GBP C-terminal genetic variants provide a fitness advantage to the host in the face of pathogen antagonism, evolutionary theory predicts that such variants could rapidly and repeatedly spread through host populations due to the forces of positive selection (also termed “Darwinian selection”). One method to infer instances of repeated positive selection in protein coding genes is through calculation of the ratio of nonsynonymous substitutions per nonsynonymous site relative to synonymous substitutions per synonymous site, referred to as *dN*/*dS* or ω. An elevated *dN*/*dS* ratio value of greater than 1 indicates that amino acid substitutions have become fixed in populations more rapidly than would be expected to occur by chance, consistent with positive selection acting preferentially on beneficial mutations (21). To detect potential signatures of positive selection in GBP1 and GBP2, we compiled gene orthologs from simian primates by direct Sanger sequencing of cDNA from primate cell lines as well as from the GenBank database (Fig. 2A). We then subjected GBP1 and GBP2 data sets to phylogenetic tests, estimating *dN*/*dS* ratios at individual sites, implemented through the PAML and HyPhy software packages (22, 23) (see Materials and Methods). All tests identified statistically significant support for positive selection acting on both GBP1 (Fig. 2B) (Fig. S1; see also Tables S1 and S2) and GBP2 (Fig. 2C) (Fig. S2; see also Tables S3 and S4). Notably, multiple positions in the C-terminal regions of both GBP1 and GBP2 exhibited signatures of positive selection, whereas the adjacent CaaX box was found to be highly conserved. We observed that the highest degree of variation in these sites appears to be present in New World primates, which diverged from the common ancestor of humans roughly 40 million years ago. These findings suggest that both GBP1 and GBP2 have been subject to repeated positive selection in the primate lineage, including at sites in the PBM which promote intracellular pathogen recognition.

10.1128/mBio.00340-20.6 TABLE S1 GBP1 whole-gene log likelihood scores and parameter estimates for four models of variable ω among sites, assuming the f3X4 model of codon frequencies in PAML. Download Table S1, PDF file, 0.02 MB. Copyright © 2020 Kohler et al. 2020 Kohler et al. This content is distributed under the terms of the Creative Commons Attribution 4.0 International license.

10.1128/mBio.00340-20.7 TABLE S2 (A) Summary of tests for positive selection in primate GBP1 (HyPhy). (B) Summary of tests for positive selection in primate GBP1 using FUBAR algorithm. (C) Summary of positive selection in primate GBP1 using REL algorithm. Download Table S2, PDF file, 0.03 MB. Copyright © 2020 Kohler et al. 2020 Kohler et al. This content is distributed under the terms of the Creative Commons Attribution 4.0 International license.

10.1128/mBio.00340-20.8 TABLE S3 GBP2 whole-gene log likelihood scores and parameter estimates for four models of variable ω among sites, assuming the f3X4 model of codon frequencies (PAML). Download Table S3, PDF file, 0.03 MB. Copyright © 2020 Kohler et al. 2020 Kohler et al. This content is distributed under the terms of the Creative Commons Attribution 4.0 International license.

### The variable polybasic motif of primate GBP1 modulates targeting of pathogenic Shigella flexneri.

**FIG 3:**
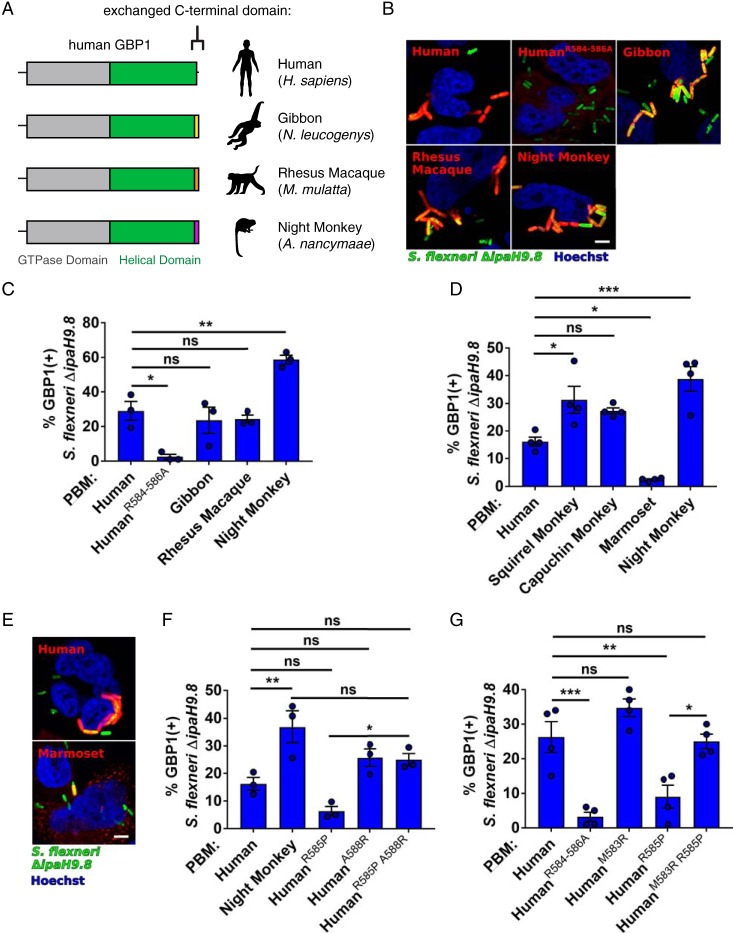
Diversification of the GBP1 polybasic motif in primates enhances recognition of Shigella flexneri. (A) Diagram illustrating gene chimeras in which the human GBP1 C terminus was replaced with corresponding sequences from related primate species. These mCherry-tagged constructs were expressed in *GBP1*^KO^ HeLa cells for subsequent experiments. (B) Representative fluorescence images of *GBP1*^KO^ cells expressing mCherry-GBP1 chimeras (red) infected with GFP-expressing S. flexneri Δ*ipaH9.8* strain (green). Hoechst stain of DNA is shown in blue. (C) Quantification of intracellular S. flexneri colocalizing with mCherry-GBP1 chimeras. Bar graphs show means ± standard errors of the means (SEM) of results from three independent experiments. (D) Quantification of GBP1-S. flexneri colocalization using New World monkey GBP1 chimera constructs. Bar graphs show means ± SEM of results from four independent experiments. (E) Representative fluorescence images comparing human GBP1 and marmoset GBP1 chimera. (F) Quantification of GBP1-S. flexneri colocalization using human GBP1, night monkey GBP1 chimera, and single and double point mutations at sites 585 and 588 in human GBP1. Bar graphs show means ± SEM of results from three independent experiments. (G) Quantification of GBP1 PBM mutant colocalization with the S. flexneri. Bar graphs show means ± SEM of results from four independent experiments. Significance for all experiments was determined by one-way analysis of variance (ANOVA) followed by Tukey’s multiple-comparison test. *, *P* ≤ 0.05; **, *P* ≤ 0.01; ***, *P* ≤ 0.001; ns, nonsignificant.

We next sought to determine how rapid divergence in the PBM impacts pathogen recognition function of GBP1. We generated a series of protein chimeras in which the C-terminal PBM of hGBP1 (576 QDLQTKMRRRKACTIS 592) was replaced with the orthologous sequence from other primate GBP1 alleles (Fig. 3A). Human GBP1 and the chimeric constructs were expressed using an anhydrotetracycline (aTc)-inducible system in CRISPR-engineered hGBP1-deficient (knockout) HeLa cells (*GBP1*^KO^) to ensure that any targeting activity observed was due to variation in exogenously expressed GBPs. To assess the consequences of GBP1 function, we chose S. flexneri as a model pathogen given that it is targeted specifically by hGBP1 and its dissemination within the host is highly sensitive to GBP1 recruitment (11–13). Cells were infected with GFP-expressing wild-type S. flexneri strain 2457T or the coisogenic Δ*ipaH9.8* mutant. Consistent with previous results (11–13), we found that hGBP1 targeting to bacteria was dependent on the triple-arginine stretch of its PBM and was blocked by the S. flexneri hGBP1 antagonist IpaH9.8 (Fig. S3). To avoid confounding results related to IpaH9.8 antagonism of GBPs, we conducted all subsequent experiments comparing the targeting efficiencies of GBP variants using the Δ*ipaH9.8* mutant. For our initial studies, we generated chimeras using C-terminal domains from a single representative hominoid (white-cheeked gibbon, Nomascus leucogenys), Old World monkey (rhesus macaque, Macaca mulatta), and New World monkey (Ma’s night monkey, Aotus nancymaae) as well as the triple-arginine PBM mutant hGBP1^R584–586A^ as a negative control. Performing these experiments with chimeric proteins allowed us to control for interspecific sequence differences outside the C-terminal region of GBP1. These experiments revealed that despite significant sequence divergence, the C-terminal domains of gibbon, rhesus macaque, and night monkey were all capable of targeting cytosolic S. flexneri (Fig. 3B). In fact, we observed that the night monkey GBP1 C-terminal amino acid stretch possesses significantly enhanced targeting ability relative to hGBP1 (Fig. 3C). aTc-induced protein expression levels were comparable across all GBP1 chimeras and mutants in the absence or presence of S. flexneri infections (Fig. S4 and S5A), suggesting that the enhanced targeting of the night monkey C terminus was a result of specific amino acid substitutions. To further explore the consequences of GBP1 diversity in other New World primates, we generated additional chimeras using squirrel monkey (Saimiri boliviensis), capuchin (Cebus capucinus *imitator*), and marmoset (Callithrix jacchus) GBP1. Both squirrel monkey and capuchin GBP1 C-terminal motifs also displayed improved GBP1 targeting relative to human (Fig. 3D; see also Fig. S5B). In contrast, the marmoset GBP1 chimeric protein associated poorly with cytosolic S. flexneri (Fig. 3D and E). This reduced function might have been the result of an insertion of a stretch of five neutral, mostly hydrophobic amino acids (NVFFP) into the PBM of marmoset GBP1, which is not present in other primates (Fig. S1). Collectively, these results demonstrate that the ability to target intracytosolic S. flexneri has been enhanced and lost in distinct lineages of New World primates.

10.1128/mBio.00340-20.3 FIG S3 Quantification of intracellular S. flexneri colocalizing with mCherry-GBP1. Experiments were performed using wild-type (wt) S. flexneri or a mutant strain containing a deletion of the IpaH9.8 effector which targets GBP1 for degradation. Bar graphs show means ± SEM of results from three independent experiments. Significance was determined by two-way ANOVA with Tukey’s multiple-comparison test. **, *P* ≤ 0.01. Download FIG S3, PDF file, 0.2 MB. Copyright © 2020 Kohler et al. 2020 Kohler et al. This content is distributed under the terms of the Creative Commons Attribution 4.0 International license.

10.1128/mBio.00340-20.4 FIG S4 HeLa or GBP1-KO HeLa cells complemented with pInducer-mCherry-GBP1 constructs were stimulated overnight with either 200 U/ml gamma interferon (IFN-γ) (HeLa + IFN-γ controls) or 0.5 μg/ml aTc. The following day, cells were left uninfected or infected with wild-type (WT) S. flexneri at an MOI of 50 for 3 h. Western blots detecting GBP1 or GAPDH (glyceraldehyde-3-phosphate dehydrogenase) loading control are indicated. Download FIG S4, PDF file, 1.4 MB. Copyright © 2020 Kohler et al. 2020 Kohler et al. This content is distributed under the terms of the Creative Commons Attribution 4.0 International license.

10.1128/mBio.00340-20.5 FIG S5 (A to C) Western blot from GBP1 and GBP1 chimera-expressing cells. mCherry antibody was used to detect individual GBP1 protein expression. GAPDH expression was included as a loading control. Download FIG S5, PDF file, 2.5 MB. Copyright © 2020 Kohler et al. 2020 Kohler et al. This content is distributed under the terms of the Creative Commons Attribution 4.0 International license.

### Genetic interactions constrain evolution of the GBP1 polybasic motif.

To gain a more detailed understanding of how natural selection has shaped the evolution of GBP1 function, we focused on two positions corresponding to R585 and A588 in hGBP1 that exhibit signatures of repeated positive selection across primates. We initially hypothesized that mutating each position in human GBP1 to the corresponding amino acid in night monkey GBP1 might be sufficient to improve bacterial recognition activity. We introduced single amino acid substitutions at both sites in hGBP1 to amino acids found in night monkey GBP1 and generated a double mutant protein. Negating our initial hypothesis, substitution of arginine at position 585 to proline (R585P) did not improve but instead reduced human GBP1 binding to S. flexneri (Fig. 3F and G). Substituting alanine 588 to arginine (A588R) did not significantly alter targeting efficiency but significantly reversed the targeting defect resulting from the introduction of the R585P mutation into this background (Fig. 3F and G). To better define functional constraints on the PBM amino acid sequence, we then asked whether the partial loss of function caused by the R585P mutation could be suppressed by mutations other than those that introduce a positive charge at position 588 (i.e., A588R). We therefore changed the hydrophobic residue closest to position 585, i.e., the methionine residue in position 583, to an arginine (M583R). Addition of a single arginine to the hGBP1 PBM (M583R) did not significantly increase recognition of S. flexneri. However, addition of this arginine was able to restore binding in the hGBP1 R585P mutant background (Fig. 3G). These data suggest that the targeting defect caused by the R585P mutation is due to the loss of the arginine residue rather than the proline insertion and can therefore be suppressed by placing an arginine in either position 583 or 588. Collectively, these results illustrate that, despite their rapid divergence, intramolecular epistasis between sites in the PBM constrains the available evolutionary trajectories that maintain antibacterial function. Our findings also indicate that there are additional sequence features in the New World monkey PBM beyond positions 585 and 588 that contribute to its enhanced bacterial targeting relative to hGBP1.

### Polybasic motif-dependent recognition of diverse Gram-negative bacteria by primate GBP1.

**FIG 4:**
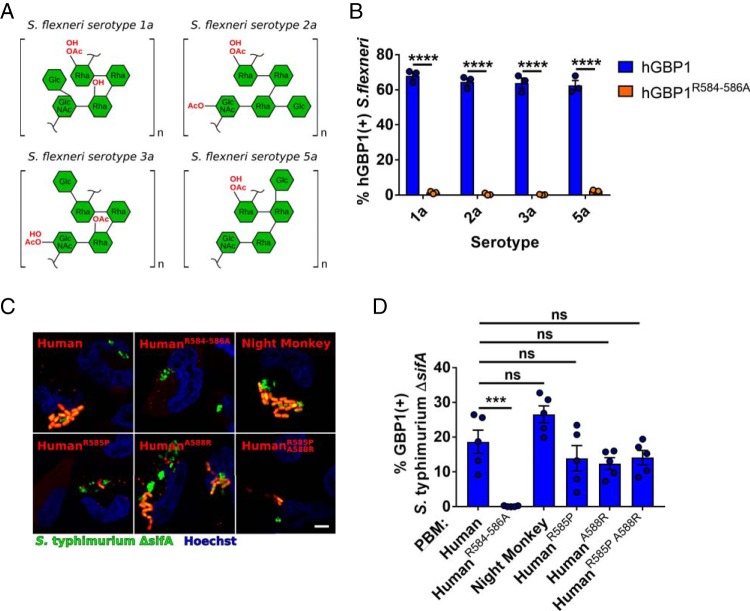
Polybasic motif-dependent recognition of Gram-negative bacteria by primate GBP1 variants. (A) O-antigen structures present in distinct *Shigella* serotypes. (B) Quantification of S. flexneri colocalization with human GBP1 (hGBP1) or the PBM GBP1 mutant (hGBP1^R584–586A^). *Shigella* serotypes encoding unique O-antigens depicted in panel A are indicated. Bar graphs show means ± SEM of results from three independent experiments. Significance was determined two-way ANOVA with Tukey’s multiple-comparison test. ****, *P* ≤ 0.0001. (C) Representative immunofluorescence images depicting colocalization of GFP-expressing *S.* Typhimurium Δ*sifA* (green) with mCherry-GBP1 chimeras (red). Hoechst stain of DNA is shown in blue. (D) Quantification of GBP1 colocalization with the *S.* Typhimurium Δ*sifA* mutant. Bar graphs show means ± SEM of results from five independent experiments. Significance was determined by one-way ANOVA followed by Tukey’s multiple-comparison test. ***, *P* ≤ 0.001; ns, nonsignificant.

We next considered whether variation between distinct bacterial populations could modulate differences in GBP1 PBM recognition between primates. Repeated episodes of positive selection acting on positions in the GBP1 PBM would suggest that intracellular pathogens are differentially targeted by GBP1 variants, leading to variable selective pressures over time. We observed that a variety of S. flexneri serotypes encoding unique O-antigen structures are similarly recognized by human GBP1 and that this recognition is dependent on the presence of the PBM (Fig. 4A and B). To assess how GBP1 diversity impacts recognition of Gram-negative pathogens beyond *Shigella*, we measured colocalization of GBP1 variants in cells infected with the Salmonella enterica Typhimurium Δ*sifA* mutant, which escapes the host phagosome to replicate in the cytoplasm (24, 25). *S.* Typhimurium Δ*sifA*, like *Shigella*, was recognized by GBP1 in a PBM-dependent manner (Fig. 4C and D). However, we noted that the level of recognition by night monkey GBP1, while appearing to be higher than that seen with human GBP1, was not significantly elevated as observed for S. flexneri (Fig. 3C and D and 4C and D). These results indicate that while the PBM is necessary for recognition of other Gram-negative bacteria, different bacterial species exhibit different levels of susceptibility to primate GBP1 orthologs. Collectively, these results illustrate how variation across primates and bacteria modulates GBP1 recognition with cytosolic pathogens.

### Convergent evolution of bacterial recognition by squirrel monkey GBP2.

**FIG 5:**
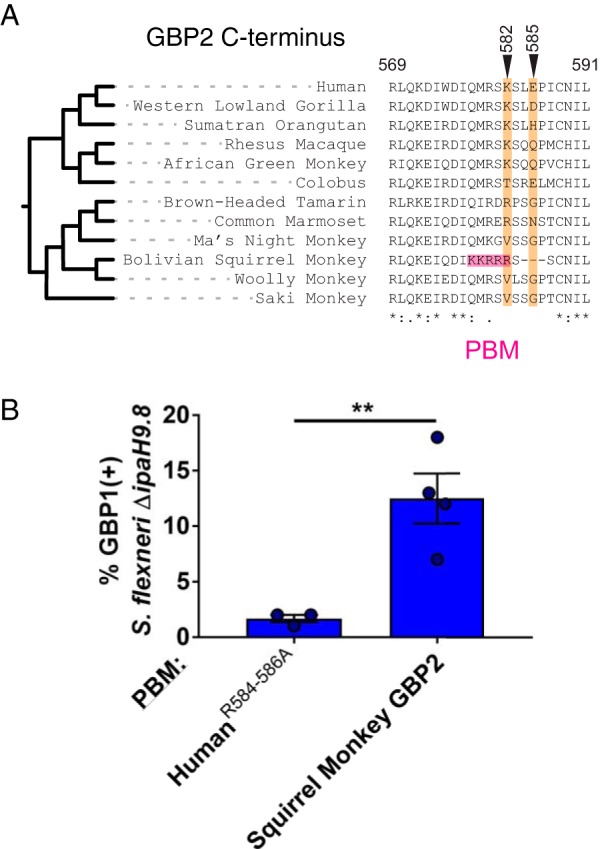
Convergent evolution of bacterial recognition by a squirrel monkey GBP2 polybasic motif. (A) Amino acid alignment of the C-terminal regions of primate GBP2 orthologs. Sites exhibiting signatures of positive selection across species are denoted in orange. The pink region highlights the emergence of a polybasic motif in squirrel monkey GBP2. (B) Quantification of GBP-S. flexneri colocalization using the hGBP1-PBM mutant and a chimeric hGBP1 fused to the C-terminal region of squirrel monkey GBP2. Bar graphs show means ± SEM of results from four independent experiments. Significance was determined by unpaired two-tailed *t* tests. **, *P* ≤ 0.01.

Similarly to hGBP1, human GBP2 (hGBP2) undergoes prenylation via its conserved C-terminal CaaX box. hGBP2 can colocalize with cytosolic S. flexneri through heterotypic interactions with hGBP1 but fails to target S. flexneri in *GBP1*^KO^ cells due to the lack of an appropriate C-terminal targeting motif (11–13). Our earlier phylogenetic analyses revealed that prenylated GBP paralogs are highly divergent at the unstructured C-terminal region immediately preceding the CaaX box, suggesting that unique C-terminal sequences direct individual prenylated hGBP isoforms toward distinct microbial targets (Fig. 2B and C). According to this model, we expect that the C-terminal residues of prenylated GBPs could be subject to conflict with intracellular pathogens evolving to evade recognition. This is consistent with the high degree of divergence among the C termini of GBP2 in primates (Fig. S2), suggesting that its microbial targeting specificity has undergone shifts during recent primate evolution. In particular, we observed that the C terminus of GBP2 in squirrel monkeys contains a series of substitutions as well as a small deletion resulting in a sequence that closely resembles the GBP1 PBM (Fig. 5A). This sequence was both observed in the publicly available Bolivian squirrel monkey (*Saimiri boliviensis*) genome and confirmed by direct Sanger sequencing of *GBP2* from the related common squirrel monkey (Saimiri sciureus). The C-terminal sequence of GBP2 from capuchin monkeys, close relatives of squirrel monkeys, was highly divergent, suggesting that these alterations arose recently in the *Saimiri* lineage (Fig. 5A). To determine if the squirrel monkey GBP2 PBM is sufficient to promote targeting of intracellular bacteria, we replaced the C-terminal region of human GBP1 with that of squirrel monkey GBP2. We observed that the resulting GBP1-GBP2 chimeric protein conferred the ability to localize to intracellular S. flexneri (Fig. 5B). This finding indicates that squirrel monkey GBP2 may have gained the ability to target intracellular Gram-negative bacteria through an example of recent convergent evolution.

## DISCUSSION

GBPs continue to emerge as critical mediators of vertebrate cell-autonomous immunity, contributing to resistance against diverse pathogens as well as susceptibility to inflammatory disease. Although GBPs exhibit variation in gene copy number and amino acid identity within and between host species, the consequences of such genetic variation for GBP function have remained unclear. The present report illustrates how rapid evolution of the C-terminal PBM in GBP1 and GBP2 controls detection of cytosol-invading pathogenic bacteria in humans and related simian primates. These findings are consistent with a model in which beneficial PBM mutations that enhance pathogen recognition have rapidly spread through host populations by the process of positive selection. The observed patterns of repeated selection in GBP1 and GBP2 could reflect two scenarios, the first being a classic “arms race” in which GBPs and specific bacterial surface molecules antagonistically coevolve to improve and impair recognition of bacterial surfaces, respectively. These patterns could also arise from fluctuations in the types of pathogens that have imposed selection on host populations over time, with PBM mutations altering the spectrum of targets recognized by a particular GBP. It is possible that both scenarios have influenced the GBP family during vertebrate evolution. While our studies have focused on host-S. flexneri interactions as a model system, we expect that PBM variation has impacted GBP activity directed toward a range of cytosolic bacteria during the course of vertebrate divergence. The diversification of GBP pathogen-targeting capabilities is also highly relevant to animal models of infection as the mouse ortholog of hGBP1, mouse GBP2, detects cytosolic S. flexneri through a poorly defined process (13) independently of a bona-fide PBM (26). Given the dynamic changes observed in a subset of simian primates, it is likely that GBPs from other nonmodel vertebrates could harbor as-yet-undiscovered pathogen-targeting capabilities.

The evolution-guided experimental approach applied here provided new details regarding the molecular basis of GBP target recognition. The high degree of conservation in GBP1 and GBP2 CaaX boxes suggests that posttranslational prenylation and subsequent membrane association have been critical for the function of both proteins. These findings agree with numerous studies that illustrated how these GBPs are able to associate with diverse intracellular membranes that include pathogen-containing vacuoles, such as those occupied by the bacterium *Chlamydia* or the protist *Toxoplasma*, was well as viral replication complexes and bacterial cell envelopes (5, 27, 28). By first associating with these target membranes, GBP1 and GBP2 are able to recruit other GBP family members as well as additional immune effectors such as the immunity-related GTPases (29). In contrast to CaaX box conservation, dynamic evolution of the adjacent PBM is indicative of selective pressures to target rapidly diversifying pathogen targets. Our results suggest that the PBM could function as an intracellular “Zip code,” allowing GBPs to distinguish between nonself and self membrane surfaces (18). In this respect, it is also of note that the CaaX box and PBM are present in a wide range of GTPases that do not perform primary roles in cell-autonomous immunity, including Rab and Rho (30). It is thus tempting to speculate that ancestral interferon-stimulated GTPase function may have emerged from a more promiscuous prenylated GTPase which evolved to perform intracellular housekeeping functions. This model is consistent with our recent finding that both hGBP1 and mouse GBPs are able to detect vacuolar membrane damage and to intersect with the galectin protein family involved in the removal of damaged organelles (31). Future evolution-guided molecular studies could aid in understanding the types of PBM-substrate interactions that underlie the diversity of cellular functions that depend on CaaX-proximal PBMs. In this regard, the evolution of the GBP PBM resembles a model of evolutionary “tinkering” proposed by François Jacob (32) and observed in other cases of protein diversification (33). Among closely related primates, we observe instances of enhanced targeting ability in New World monkeys but also cases where new mutations have attenuated GBP function such as is seen with marmoset GBP1. Mutation of individual sites in the PBM of GBP1 further demonstrated that epistasis could strongly constrain evolutionary paths to new functions. The fact that vertebrate genomes often encode several GBP family members with cooperative and overlapping targeting abilities may relax selective constraint on single GBP genes to allow for this exploration of broader sequence space. GBPs therefore provide an attractive and tractable model to investigate fundamental issues concerning evolutionary novelty.

Much work delineating the molecular mechanisms of host-microbe genetic conflict has focused on interactions with viruses (34–40), although emerging studies performed by ourselves and others have highlighted the potential for pathogenic bacteria to promote similar evolutionary dynamics (41–43). Given the ability of GBPs to target a diverse array of pathogens and pathogen-containing compartments, future studies aimed at understanding potential trade-offs in target specificity during GBP evolution would greatly improve our understanding of their functions in cell-autonomous immunity. The ability of GBPs to also cooperate and form heteromeric complexes likely provides combinatorial breadth in pathogen recognition.

In addition to the investigations of GBP family evolution reported here, previous work established that members of the myxovirus resistance (Mx) protein family of interferon-stimulated GTPases have also been subject to repeated positive selection in primates (44, 45) as well as to counteradaptation by viral pathogens (46). While GBP and Mx protein family GTPases differ in their molecular targets and the specific mechanism by which pathogen recognition occurs, they may share fundamental principles underlying their immune surveillance functions. Mammalian Mx protein diversity, particularly within the L4 loop of the alpha-helical stalk region, controls the breadth and specificity of the viral proteins recognized by this restriction factor (44, 47, 48). The combination of Mx protein oligomerization and L4 loop flexibility could provide a broad target interface to mediate the interaction of these antiviral GTPases with diverse viral protein substrates (49). We propose that, similarly to the L4 loop of Mx proteins, the unstructured C-terminal regions preceding the CaaX boxes of GBP1, GBP2, and GBP5 confer target specificities and equip these prenylated proteins with the ability to associate with pathogen membranes or pathogen-containing membrane-bound compartments. Parallels between the evolution of the PBM of GBPs and the L4 loop of MxA are indicative of diverse intracellular pathogens exerting selective pressure on both protein families across different host species. A recent study of MxA diversity further highlighted the potential for trade-offs between the breadth and the specificity of antiviral activity during the evolution of the L4 loop (50). Such observations are consistent with both dynamic changes in copy number and sequence variation of interferon-stimulated GTPases occurring in order to target diverse pathogens.

Recent work indicated that S. flexneri encodes a secreted effector protein, IpaH9.8, which targets GBPs for degradation by the proteasome (12, 13). Although our preliminary studies did not reveal any significant differences in the abilities of IpaH9.8 to antagonize GBP variants, it is entirely possible that other microbial GBP inhibitors also imposed selective pressure on this gene family during animal evolution. In support of this hypothesis, our phylogenetic analyses identified signatures of positive selection acting on sites beyond the PBM, namely, in the GTPase and alpha-helical domains of GBP1 and GBP2 (Fig. 2B and C). Future experiments may resolve if and how GBP evolution impacts resistance to other, as-yet-to-be-discovered pathogen-encoded inhibitors. Together this study establishes functional links between GBP evolution and the molecular basis of intracellular bacterial pathogen recognition.

## MATERIALS AND METHODS

### Primate GBP genetic sources.

Primate *GBP1* and *GBP2* sequences were retrieved from NCBI GenBank entries for primates with sequenced genomes. For other primates, sequences were obtained by Sanger sequencing of PCR amplicons using cDNA isolated from primary cell lines obtained from Coriell Cell Repositories (Camden, NJ). Briefly, RNA was harvested using a ZR-*Duet* DNA/RNA MiniPrep Plus kit (Zymo Research). Isolated RNA (50 μg) from cell lines was used as a template for reverse transcriptase PCR (RT-PCR) (SuperScript III; Invitrogen). Sequences of interest were PCR amplified from cDNA using Phusion High-Fidelity master mix (Thermo) according to the manufacturer’s protocol and were cloned into pCR2.1 (Invitrogen). Sanger sequencing was performed from at least three individual clones. *GBP1* and *GBP2* gene sequences obtained from the NCBI database included human (Homo sapiens), chimpanzee (Pan troglodytes), bonobo (Pan paniscus), Western lowland gorilla (Gorilla gorilla), Sumatran orangutan (Pongo abelii), sooty mangabey (Cercocebus atys), drill (Mandrillus leucophaeus), olive baboon (Papio anubis), Rhesus macaque (Macaca mulatta), crab-eating macaque (Macaca fascicularis), pit-tailed macaque (Macaca nemestrina), green monkey (Chlorocebus sabaeus), colobus (Colobus angolensis *palliatus*), common marmoset (Callithrix jacchus), Ma’s night monkey (Aotus nancymaae), capuchin monkey (Cebus capucinus *imitator*), and Bolivian squirrel monkey (Saimiri boliviensis). The *GBP1* orthologs cloned from cDNA (with Coriell identifier [ID] numbers) are as follows: white-handed gibbon (PR01121), white-cheeked gibbon (PR00712), red-chested mustached tamarin (AG05308), saddleback tamarin (AG05313), and common woolly monkey (AG05356). The *GBP2* orthologs cloned from cDNA (with Coriell ID numbers) are as follows: patas monkey (AG06116), red-chested mustached tamarin (AG05308), common squirrel monkey (AG05311), common woolly monkey (AG05356), and white-faced saki (PR00239). GBP gene sequence data from this project has been deposited in GenBank under accession numbers MT262957 to MT262966.

### GBP phylogenetic and protein structure analysis.

DNA multiple-sequence alignments were performed using MUSCLE, and indels were manually edited based on amino acid comparisons. Phylogenetic trees for each sequence set were derived from consensus primate species relationships (51). Maximum-likelihood analyses of the *GBP1* and *GBP2* data sets were performed with codeml of the PAML software package (22). Positive selection was assessed by fitting the multiple alignment to either F3X4 or F61 codon frequency models. Likelihood ratio tests (LRTs) were performed by comparing the site-specific models (NS sites) as follows: M1 (neutral) was compared with M2 (selection) and M7 (neutral, beta distribution of *dN*/*dS* < 1) with M8 (selection, beta distribution of *dN*/*dS* > 1 allowed). PAML identified sets of amino acids with high posterior (greater than 0.95) probabilities for positive selection by a Bayesian approach. Additional LRTs from the HyPhy software package which account for synonymous rate variation and recombination (FEL, SLAC, and MEME) were performed using the Datamonkey server (23). Sites under positive selection for GBP1 were mapped onto three-dimensional molecular structures available from the Protein Data Bank (PDB ID 1DG3) using Chimera (52) (https://www.cgl.ucsf.edu/chimera). GBP2 sites of positive selection were mapped onto a three-dimensional molecular structure generated using the I-Tasser modeling program provided by the University of Michigan (https://zhanglab.ccmb.med.umich.edu/I-TASSER).

### Design of GBP expression constructs.

Plasmids encoding mCherry-tagged hGBP1 and a triple arginine mutation in the hGBP1 PBM were previously reported (11). The mCherry-tagged hGBP1 plasmids were used as a template to generate hGBP1-primate GBP chimeras. First, a BglII restriction site within the linker sequence separating the N-terminal mCherry-tag from hGBP1 was eliminated in pmCherry-hGBP1 by QuikChange site-directed mutagenesis (Agilent) using the oligomer pair pmCherry-hGBP1DBglII-F and -R (see Table S5 in the supplemental material). Next, 5′-Kozak-mCherry-hGBP1 from the resulting vector was amplified with oligomers that simultaneously added 5′-attB1 and 3′-attB2 sites and introduced a BglII site spanning hGBP1 codons Q577 to L579 via a synonymous mutation in codon Q577 (CAG to CAA) and truncated GBP1 beyond codon L579 (attB1-mCherry-F and attB2-hGBP1DC_BglII-R; Table S5). This PCR product was inserted into pDONR221 (Invitrogen) via Gateway BP recombination (Invitrogen). Sequences encoding primate PBMs were added to the resulting pDONR221-mCherry-human GBP1DC_BglII vector following BglII digestion performed using ligation-independent cloning (In-Fusion; Clontech) with annealed oligomers that also restored human GBP1 Q580 to L581 and the human GBP1 CaaX box (Table S5). Finally, the resulting chimeras were inserted into lentiviral tetracycline-inducible vector pInducer20 (53) by Gateway LR recombination (Invitrogen).

10.1128/mBio.00340-20.9 TABLE S4 (A) Summary of positive selection in primate GBP2 (MEME, FEL, SLAC). (B) Summary of positive selection in primate GBP2 using REL algorithm. (C) Summary of positive selection in primate GBP2 using FUBAR algorithm. Download Table S4, PDF file, 0.02 MB. Copyright © 2020 Kohler et al. 2020 Kohler et al. This content is distributed under the terms of the Creative Commons Attribution 4.0 International license.

10.1128/mBio.00340-20.10 TABLE S5 Oligonucleotides used in this study. Download Table S5, PDF file, 0.02 MB. Copyright © 2020 Kohler et al. 2020 Kohler et al. This content is distributed under the terms of the Creative Commons Attribution 4.0 International license.

5′-Kozak-mCherry-hGBP1 was cloned into pDONR221 by Gateway BP recombination following amplification with primers attB1-mCherry-F and attB2-hGBP1-R, followed by insertion into pInducer20 by Gateway LR recombination. Mutant R585P and A588R alleles were constructed from pDONR221-mCherry-human GBP1 by QuikChange site directed mutagenesis using oligomer pairs hGBP1_R585P-F and -R, hGBP1_A588R-F and -R, and hGBP1_R585P_A588R-F and -R (Table S5). The resulting mutant constructs were inserted into pInducer20 via Gateway LR recombination.

To construct the chimera in which the C-terminal portion of hGBP1 was replaced with that of Bolivian squirrel monkey GBP2, a derivative of pmCherry-human GBP1 was used in which a synonymous mutation was made within the flexible region separating alpha-helices 11 and 12 to introduce a BclI restriction enzyme site. This plasmid was propagated in Escherichia coli lacking *dam*/*dcm* (New England Biolabs), and a synthetic “gBlock” encoding Bolivian squirrel monkey GBP2 residues 576 to 588 was inserted via BclI restriction digest/ligation.

### Cell lines, cell culture, and ectopic gene expression.

hGBP1-deficient HeLa cells (*GBP1*^KO^) were described previously (11). Unless noted otherwise, *GBP1*^KO^ cells were stably transduced with an aTc-inducible gene expression systems to drive the expression of hGBP1 as well as that of its mutant and chimera variants. For transient-transfection experiments, cells were transfected with the indicated expression constructs using Lipofectamin LTX (Thermo Fisher Scientific). Cells were cultivated in Dulbecco’s modified Eagle medium (Gibco, Thermo Fisher Scientific) supplemented with 10% fetal bovine serum (Corning), 1% nonessential amino acids (Sigma), and 55 μM β-mercaptoethanol (Gibco, Thermo Fisher Scientific) at 37°C and 5% CO_2_.

### Bacterial strains and infections.

*GBP1^KO^* HeLa cells were cultured on glass coverslips and infected with GFP-expressing S. flexneri strain 2547T or the coisogenic Δ*ipaH9.8* GFP-positive (GFP^+^) mutant strain at a multiplicity of infection (MOI) of 50, essentially as described previously (11), or with *S.* Typhimurium Δ*sifA* GFP^+^ at an MOI of 25. Briefly, tryptic soy broth (TSB) or Luria broth (LB)-Miller supplemented with 50 μg/ml carbenicillin or 30 μg/ml kanamycin was inoculated with a single colony, in the case of S. flexneri Congo red-positive results, and grown overnight at 37°C with shaking. Stationary overnight cultures were diluted 1:30 in 5 ml of fresh TSB or 1:33 in 1 ml fresh LB and incubated for 1 h to 1.5 h at 37°C with shaking until an optical density at 600 nm (OD_600_) of 0.4 to 0.6 was reached or for 2 h 40 min with shaking until an OD_600_ of 1.6 to 2.0 was reached. Bacteria were diluted in prewarmed cell culture medium and spun onto host cells for 10 min at 700 × *g*. Infected cells were incubated for 30 min at 37°C and 5% CO_2_ and subsequently washed twice with Hanks balanced salt solution (HBSS), followed by addition of cell culture medium containing 25 mg/ml gentamicin. Cells were incubated for an additional 2.5 h or 3.5 h at 37°C and 5% and then fixed in 4% paraformaldehyde for 15 min at room temperature and mounted onto glass slides for fluorescence microscopy. Fixed cells were imaged using a Zeiss Axio Observer.Z1 microscope, and image analysis was performed to quantify colocalization of mCherry fusion proteins with bacteria as described previously (11). Confocal images were taken with a Zeiss 880 Airyscan inverted microscope.

### Data accessibility.

GBP gene sequence data from this project have been deposited in GenBank under accession numbers MT262957 to MT262966.
