# Supplementary material for: A Rapidly Evolving Polybasic Motif Modulates Bacterial Detection by Guanylate Binding Proteins
Source: mBio. 2020 May 19;11(3):e00340-20. doi: 10.1128/mBio.00340-20 (PMC7240152; doi:10.1128/mBio.00340-20)
Supplement: FIG S1 [file mBio.00340-20-sf001.pdf]

```
                                             210
                        192                    ▼                     234
                  Human DGQPLTPDEYLTYSLKLKKGTSQKDETFNLPRLCIRKFFPKKK
                 Bonobo DGQPLTPDEYLTYSLKLKKGTSQKDETFNLPRLCIRKFFPKKK
             Chimpanzee DGQPLTPDEYLTYSLKLKKGTSQKDETFNLPRLCIRKFFPKKK
 Western Lowland Gorilla DGQPLTPDEYLTYSLKLKKGTSQKDETFNLPRLCIRKFFPKKK
      Sumatran Orangutan DGQPLTPDEYLTYSLKLKKGTSQKDETFNLPRLCIRKFFPKKK
     White-Handed Gibbon DGQPLTPDEYLTYSLKLKKGTSEKDKTFNLPRLCIRKFFPKKK
    White-Cheeked Gibbon DGQPLTPDEYLTYSLKLKKGTSEKDKTFNLPRLCIRKFFPKKK

                Mangabey DGQPITADEYLTYSLKLKHGTSEKDKTFNLPRLCIRKYFPKKK
                   Drill DGQPITADEYLTYSLKLKQGTSEKDKTFNLPRLCIRKFFPKKK
                  Baboon DGQPITADEYLTYSLKLKQGTSEKDKTFNLPRLCIRKYFPKKK
          Rhesus Macaque DGQPITADEYLTYSLKLKQGTSEKDKNFNLPRLCIRKFFPKKK
      Crab-Eating Macaque DGQPITADEYLTYSLKLKQGTSEKDKNFNLPRLCIRKFFPKKK
       Pigtailed Macaque DGQPITADEYLTYSLKLKQGTSEKDKNFNLPRLCIRKFFPKKK
     African Green Monkey DGQPITADEYLTYSLKLKKGTSEKDKTFNLPRLCIRKFFPKKK
Golden Snubbed-Nosed Monkey DGQPITADEYLTYSLKLKQGTSEKDKNFNLPRLCIRKFFPKKK
                 Colobus DGQPITADEYLTYSLKLKQGTSEKDKTFNLPRLCIRKFFPKKK

    Brown-Headed Tamarin NGQPITADEYLEYSLKLKQGTSQKDKNFNLPRLCIRKFFPKKK
Red-Chested Mustached Tamarin HGQPITADEYLEYSLKLKKGTSEKDKNFNLPRLCIRKFFPKKK
         Common Marmoset NGQPITADEYLEYSLKLKPGTSQKDKNFNLPRLCIRKFFPKKK
        Ma's Night Monkey NGQPITADEYLEYSLKLKQVTSQKDKNFNLPRLCIRKFFPKKK
  Bolivian Squirrel Monkey NGVPITADEYLEYSLKLKQGTSQKDKNFNLPRLCIRKFFPKKK
         Capuchin Monkey NGVPITADEYLEYSLKLKQDTSQKDKNFNLPRLCIRKFFPKKK
           Woolly Monkey NGKPITADEYLEHSLKLKQGTSQKDKNFNLPRLCIRKFFPKKK
                          * *:*.**** :*****  **:**:.**********:*****
```

```
                                                264
                        250                      ▼                        295
                  Human LEKLQDEELDPEFVQQVADFCSYIFSNSKTKTLSGGIQVNGPRLES
                 Bonobo LEKLQDEELDPEFVQQVADFCSYIFSNSKTKTLSGGIQVNGPRLES
             Chimpanzee LEKLQDEELDPEFVQQVADFCSYIFSNSKTKTLSGGIQVNGPRLES
 Western Lowland Gorilla LEKLQDEELDPEFVQQVADFCSYIFSNSKTKTLSGGIQVNGPRLES
      Sumatran Orangutan LEKLQDEELDPEFVQQVADFCSYIFSNSKTKTLSGGIQVNGPRLES
     White-Handed Gibbon LEKLQDEELDPEFVQQVADFCSYIFSNSKTKTLSGGIKVNGPRLES
    White-Cheeked Gibbon LEKLQDEELDPEFVQQVADFCSYIFSNSKTKTLSGGIKVNGPRLES

                Mangabey LEKLHDEELDPEFVQQVADFCSYIFSNSKTKTLSGGIKVNGPRLES
                   Drill LEKLHDEELDPEFVQQVADFCSYIFSNSKTKTLSGGIKVNGPRLES
                  Baboon LEKLHDEELDPEFVQQVADFCSYIFSNSKTKTLSGGIKVNGPRLES
          Rhesus Macaque LEKLHDEELDPEFVQQVADFCSYIFSNSKTKTLSGGIKVNGPRLES
      Crab-Eating Macaque LEKLHDEELDPEFVQQVADFCSYIFSNSKTKTLSGGIKVNGPRLES
       Pigtailed Macaque LEKLHDEELDPEFVQQVADFCSYIFSNSKTKTLSGGIKVNGPRLES
     African Green Monkey LEKLHDEELDPEFVQQVADFCSYIFSNSKTKTLSGGIKVNGPRLES
Golden Snubbed-Nosed Monkey LEKLHDEELDPEFVQQVADFCSYIFSNSKTKTLSGGIKVNGPRLES
                 Colobus LEKLHDEELDPEFVQQVADFCSYIFSNSKTKTLSGGIKVNGPRLES

    Brown-Headed Tamarin LEKLHDAELDPEFVLQVTNFCSYIFTHSKTKTLSGGIKVNGPRLEI
Red-Chested Mustached Tamarin LEKLHDAELDPEFVLQVANFCSYIFTHSKTKTLSGGIKVNGPRLEI
         Common Marmoset LEKLHDEELDSEFVLQVANFCSYIFTHSKTKILSGGIKVNGPRLES
        Ma's Night Monkey LEKLHDEDLDPDFVQQVADFCSYIFTNSKTKTLSGGIKVNGPRLES
  Bolivian Squirrel Monkey LEKLHDEQLDPEFVLQVADFCSYIFTNSKTKTLSGGIKVNGPRLES
         Capuchin Monkey LEKLHDEELDTDFVQQVADFCSYIFTNSKTKTLSGGIKVNGPRLES
           Woolly Monkey LEKLHDEELDPEFVRQVADFCSYIFTSSKTKTLSGGIKVNGPRLES
                        ****:* :**.:** **::******: **** *****:*******
```

```
                                             503
                        485                   ▼                     527
                  Human KEKEIEVERVKAESAQASAKMLQEMQRKNEQMMEQKERSYQEH
                 Bonobo KEKEIEVERVKAESAQASAKMLQEMQRKNEQMMEQKERSYQEH
             Chimpanzee KEKEIEVERVKAESAQASAKMLQEMQRKNEQMMEQKERSYQEH
 Western Lowland Gorilla KEKEIEVERVKAESAQASAKMLQEMQRKNEQMMEQKERSYQEH
      Sumatran Orangutan KEKEIEVERVKAESAQASAKMLQEMQRKNEQMMEQKERSYQEH
     White-Handed Gibbon KEKEIEVERVKAESAQASAKMLQEMQRKNEQMMEQKERSYQEH
    White-Cheeked Gibbon KEKEIEVERVKAESAQASAKMLQEMQRKNEQMMEQKEKSYQEH

                Mangabey KEKEIEVERVKAESAQASTKMLQEIQRKNEQMMEQKERSYQEH
                   Drill KEKEIEVERVKAESAQASTKMLQEIQRKNEQMMEQKERSYQEH
                  Baboon KEKEIEVERVKAESAQASTKMLQEMQRKNEQMMEQKERSYQEH
          Rhesus Macaque KEKEIEVERVKAESAQASAKMLQEMQRKNEQMMEQKERSYQEH
      Crab-Eating Macaque KEKEIEVERVKAESAQASAKMLQEMQRKNEQMMEQKERSYQEH
       Pigtailed Macaque KEKEIEVERVKAESAQASTKMLQEIQRKNEQMMEQKERSYQEH
     African Green Monkey KEKEIEVERVKAESAQASTKMLQEIQRKNEQMMEQKERSYQEH
Golden Snubbed-Nosed Monkey KEKEIEVERVKAESAQASTKMLQEMQRKNEQMMEQKERSYQEH
                 Colobus KEKEIEVERVKAESAQASAKMLQEMQRKNEQMMEQKERSYQEH

    Brown-Headed Tamarin KEKDIEVERVKAESAQASTKMLEEMQRKNEQMMEQKERSYQEH
Red-Chested Mustached Tamarin KEKDIEVERVKAESAQASTKMLEEMQRKNEQMMEQKERSYQEH
         Common Marmoset KEKEIEVERVKAESAQASAKMLEEMQRKNEQMMEQKERSYQEH
        Ma's Night Monkey KEKEIEVERVKAESAQASAKMLEEMQRKNEQMMEQKERSYQEH
  Bolivian Squirrel Monkey KEKEIEVERVKAESAQASAKMLEEMQRKNEQMMEQKERSYQEH
         Capuchin Monkey KEKEIEVERVKAESAQASAKMLEEMQRKNEQMMEQKEKSYQEH
           Woolly Monkey KEKDIEVERVKAESAQASAKMLEEMQRKNEQMMEQKERSYQEH
                        ***:**************:***:*:************.*****
```

```
                                                        585 588
                        563                              ▼  ▼   592
                  Human EGFQKESRIMKNEIQDLQTKMR-------RRKACTIS
                 Bonobo EGFQKESRIMKNEIQDLQTKMR-------RRKACTIS
             Chimpanzee EGFQKESRIMKNEIQDLQTKMR-------RRKACTIS
 Western Lowland Gorilla EGFQKESRIMKNEIQNLQTKMR-------RRKACTIS
      Sumatran Orangutan EGFQKESRIMKNEIQDLQTKMR-------RRKACTIS
     White-Handed Gibbon EGFQKESRIMQNEIKDLQTKMR-------RRRPCTIS
    White-Cheeked Gibbon EGFQKESRIMQNEIKDLQAKMR-------RRRPCTIS

                Mangabey EGFQTESRKMQNEIQDLQKKMR-------QRRTCTIS
                   Drill EGFQTESRKMQNEIQDLQKKMR-------QRRICTIS
                  Baboon EGFQTESRKMQNEIQDLQKKMR-------QRRTCTIS
          Rhesus Macaque EGFQTESRKMQNEIQDLQKKMR-------QRRTCTIS
      Crab-Eating Macaque EGFQTESRKMQNEIQDLQKKMR-------QRRTCTIS
       Pigtailed Macaque EGFQTESRKMQNEIQDLQKKMR-------QRRTCTIS
     African Green Monkey EGFQTESRKMQNEIQDLQKKMR-------QRRTCTIS
Golden Snubbed-Nosed Monkey EGFQTESRKMQHEIQDLQKKMR-------QRRTCTIS
                 Colobus EGFQTESRKMQNEIQDLQKKMR-------QRRTCTIS

    Brown-Headed Tamarin EGFENESRRMQNEIRNLQNAMKKALFPSLLRRRCTIS
Red-Chested Mustached Tamarin EGFENESRRMQNEIRNLQNAMKKAFFPSLPRRRCTIS
         Common Marmoset EGFENESRRMQNEIQILRNAMKNVFFP--LSRRCTIS
        Ma's Night Monkey EGFENESRRMQNEIRNLQNAMR----P--PRRRCTIS
  Bolivian Squirrel Monkey EGFENESRRMQNEIRNLQNAMR-------PRKRCTIS
         Capuchin Monkey EGFQNESRRMQNEIRNLQNAMK----P--PRRRCTIS
           Woolly Monkey EGFENESRRMQNEIRNLQNAMR----P--PKRRCTIS
                        ***:.*** *::**. *.  *.        . ****
```
