# Supplementary material for: A Rapidly Evolving Polybasic Motif Modulates Bacterial Detection by Guanylate Binding Proteins
Source: mBio. 2020 May 19;11(3):e00340-20. doi: 10.1128/mBio.00340-20 (PMC7240152; doi:10.1128/mBio.00340-20)
Supplement: FIG S2 [file mBio.00340-20-sf002.pdf]

```
                                                    247
                           228                       ▼      255
                     Human FPKRKCFVFDWPAPKKYLAHLEQLKEEE
                    Bonobo FPKRKCFVFDWPAPKKYLAHLEQLKEEE
                Chimpanzee FPKRKCFVFDWPAPKKYLAHLEQLKEEE
   Western Lowland Gorilla FPKRKCFVFDWPAPKKYLAHLEQLKEEE
        Sumatran Orangutan FPKRKCFVFDWPAPRKYLAHLEQLKEEE

            Sooty Mangabey FPNRKCFIFDWPAQKKYLAHLEQLKEEE
                     Drill FPNRKCFIFDWPAQ-KYLARLEQLKEEE
                    Baboon FPNRKCFIFDWPAHKKYLARLEQLKEEE
            Rhesus Macaque FPNRKCFIFDWPAQKKYLARLEQLKEEE
       Crab-Eating Macaque FPNRKCFIFDWPAQKKYLARLEQLKEEE
         Pigtailed Macaque FPNRKCFIFDWPAQKKYLARLEQLKEEE
      African Green Monkey FPNRKCFIFDCPAQKKYLARLEQLKEEE
              Patas Monkey FPNRKCFIFDCPAQKKYLARLEQLKEEE
                   Colobus FPNRKCFIFDWPAQKKYLARLEQLKEEE

      Brown-Headed Tamarin FPKRKCFIFDRPARTKYLACLEQLKEED
           Common Marmoset FPKRKCFVFDRPARGKYLPHLEQLKEED
          Ma’s Night Monkey FPKRKCFTFDRPTRRKYLAHLEQLKEED
           Capuchin Monkey FPKRKCFIFDRPARRKYLAHLEQLKEED
  Bolivian Squirrel Monkey FPKRKCFIFEQPAQRTLLARLEQLKEED
               Saki Monkey FPKRKCFIFDRPARRKYLACLEQLKEED
             Woolly Monkey FPKRKCFIFDRPAQRKYLAHLEQLKEED
                           **:**** *: *:  . *. *******:
```

```
                                   550                                              582  585
                           541      ▼                                                 ▼    ▼        591
                     Human MAEQEKTLALKLQEQERLLKEGFENESKRLQKDIWDIQMRS-----KSLEPICNIL
                    Bonobo MAEQEKTLALKLQEQERLLKEGFENESKRLQKDIWDIQMRS-----KSLEPICNIL
                Chimpanzee MAEQEKTLALKLQEQERLLKEGFENESKRLQKDIWDIQMRS-----KSLEPICNIL
   Western Lowland Gorilla MAEQEKTLALKLQEQERLLKEGFENESKRLQKDIWDIQMRS-----KSLDPICNIL
        Sumatran Orangutan MAEQEKTLALKLREQERLLKEGFENESKRLQKEIRDIQMRS-----KSLHPICNIL

            Sooty Mangabey MAEQEKTLALKLQEQERLLKEGFKNESQRLQKEIRDIQMRS-----KSRQPTCHIL
                     Drill MAEQEKTLALKLQEQERLLKEGFKNESQRLQKEIRDIQMRS-----KSRQPTCHIL
                    Baboon MAEQEKTLALKLQEQERLLKEGFKNESQRLQKEIRDIQMRS-----KSRQPTCHIL
            Rhesus Macaque MAEQEKTLALKLQEQERLLKEGFKNESQRLQKEIRDIQMRS-----KSQQPMCHIL
       Crab-Eating Macaque MAEQEKTLALKLQEQERLLKEGFKNESQRLQKEIRDIQMRS-----KSQQPMCHIL
         Pigtailed Macaque MAEQEKTLALKLQEQERLLKEGFKNESQRLQKEIRDIQMRS-----KSQQPMCHIL
      African Green Monkey MAEQEKTLALKLQEQERLLKEGFKNESQRIQKEIQDIQMRS-----KSQQPVCHIL
              Patas Monkey MAEQEKTLALKLQEQERLLKEGFKNESQRIQKEIRDIQMRS-----KSQQPMCHIL
                   Colobus MAEQEKTLALKLQEQERLLKEGFKNESQRLQKEIRDIQMRS-----TSRELMCHIL

      Brown-Headed Tamarin MAEQEKIINVKFKEQELLLKEGFENESNRLRKEIRDIQIRD-----RPSGPICNIL
           Common Marmoset MAEQEKTITCKLKEQELLLKEGFQNESKRLQKEIQDIQMRE-----RSSNSTCNIL
          Ma’s Night Monkey MAEQEKTINLKLKEQERLLKEGFENESKRLQKEIRDIQMKG-----VSSGPTCNIL
           Capuchin Monkey MAEQEKTINLKLKEQERLLKEGFENESKRLQKEIRDIQMRRVDHEVKRSRPSWSTW
  Bolivian Squirrel Monkey IAEQEKTIDVKLKEQERLLKEGFELESKRLQKEIQDIKKRR-----RS---SCNIL
               Saki Monkey MAEQEMTINLKLKEQERLLKEGFENESKRLQKEIRDIQMRS-----VSSGPTCNIL
             Woolly Monkey MAEQEKTINLKLKEQERLLKEGFKNESKRLQKEIEDIQMRS-----VLSGPTCNIL
                           :****  :  *:.*** ******: **:*:.*:* **: .
```
