# Supplementary material for: A Rapidly Evolving Polybasic Motif Modulates Bacterial Detection by Guanylate Binding Proteins
Source: mBio. 2020 May 19;11(3):e00340-20. doi: 10.1128/mBio.00340-20 (PMC7240152; doi:10.1128/mBio.00340-20)
Supplement: TABLE S1 [file mBio.00340-20-st001.pdf]

Table S1. GBP1 whole gene log likelihood scores and parameter estimates for four models of variable ω among sites assuming the f3X4 model of codon frequencies in PAML.

Site Model	Parameter Estimates	Sites* with ω**>1	lnL
**M1: Neutral**	(ω_0=0) f_0=0.624<br>(ω_1=1) f_1=0.376<br>branch ω (mean)=0.376	Not allowed	-4986.95
**M2: Selection**	(ω_0=0) f_0=0.614<br>(ω_1=1) f_1=0.374<br>**(ω_2=5.70) f_2=0.012**<br>branch ω (mean)=0.440	448Y 0.955	-4981.31
**M7: β**	p=0.00500<br>q=0.00750<br>branch ω (mean)=0.400	Not allowed	-4987.21
**M8: β and ω**	p=0.01644 q= 0.02948<br>f_0=0.987<br>**ω_1=5.41 (f_1=0.013)**<br>branch ω (mean)=0.432	**210 K** 0.964<br>424 A 0.953<br>448 Y 0.986<br>**585 R** 0.976	-4981.27

*posterior probabilities >0.95 by Bayes Empirical Bayes (BEB) analysis
**ω=dN/dS
***Amino acid positions shown are for human GBP1.
