# Supplementary material for: A Rapidly Evolving Polybasic Motif Modulates Bacterial Detection by Guanylate Binding Proteins
Source: mBio. 2020 May 19;11(3):e00340-20. doi: 10.1128/mBio.00340-20 (PMC7240152; doi:10.1128/mBio.00340-20)
Supplement: TABLE S2 [file mBio.00340-20-st002.pdf]

Table S2A. Summary of tests for positive selection in primate GBP1 (HyPhy).

Model	Sites with evidence of positive selection (p-value)*
MEME	194 Q 0.013<br>203 T 0.059<br>**210 K 0.028**<br>249 Q 0.038<br>**264 Q 0.064**<br>**503 A 0.071**<br>562 K 0.067<br>582 K 0.055<br>**585R 0.035**<br>586R 0.034<br>**588A 0.088**
FEL	**210 K 0.022**<br>**264 Q 0.045**<br>**503 A 0.051**<br>**585 R 0.025**<br>**588 A 0.066**
SLAC	No positively selected sites identified

Table S2B. Summary of tests for positive selection in primate GBP1 using FUBAR algorithm.

Sites with evidence of diversifying selection*	Posterior probability β>α
**K210**	**0.987**
**Q264**	**0.921**
**A503**	**9.932**
**R585**	**0.981**
**A588**	**0.929**

*Amino acid positions shown are for human GBP1.

Table S2C. Summary of tests for positive selection in primate GBP1 using REL algorithm.

Sites with evidence of diversifying selection*	Posterior probability β>α
Y143	0.990
H150	0.991
E218	0.990
D192	0.990
Q194	0.979
T203	0.973
**K210**	**0.998**
G211	0.992
Q214	0.977
T218	0.997
F229	0.988
A248	0.975
Q249	0.993
E257	0.991

P260	0.974
E261	0.993
**Q264**	**0.999**
I332	0.993
T349	0.993
D359	0.989
E363	0.992
E389	0.994
D405	0.991
A409	0.993
V413	0.991
A424	0.965
Y447	0.990
I455	0.980
T463	0.985
E484	0.994
**A503**	**0.999**
M509	0.965
R522	0.991
N537	0.997
V540	0.973
Q559	0.996
Q566	0.994
K567	0.991
I571	0.997
Q577	0.998
L578	0.995
K582	0.994
R584	0.973
**R585**	**0.995**
R586	0.992
K587	0.991
**A588**	**0.999**

*Amino acid positions shown are for human GBP1.
