# Supplementary material for: A Rapidly Evolving Polybasic Motif Modulates Bacterial Detection by Guanylate Binding Proteins
Source: mBio. 2020 May 19;11(3):e00340-20. doi: 10.1128/mBio.00340-20 (PMC7240152; doi:10.1128/mBio.00340-20)
Supplement: TABLE S3 [file mBio.00340-20-st003.pdf]

Table S3. GBP2 whole gene log likelihood scores and parameter estimates for four models of variable ω among sites assuming the f3X4 model of codon frequencies in PAML.

Site Model	Parameter Estimates	Sites* with ω**>1	lnL
**M1: Neutral**	(ω_0=0) f_0=0.735<br>(ω_1=1) f_1=0.265<br>branch ω (mean)=0.293	Not allowed	-5061.10
**M2: Selection**	(ω_0=0) f_0=0.732<br>(ω_1=1) f_1=0.248<br>**(ω_2=6.6) f_2=0.021**<br>branch ω (mean)=0.421	**247 H 0.993**<br>575 W 0.976<br>**582 K 0.999**<br>**585 E 1.000**<br>587 I 0.975	-5037.85
**M7: β**	*p*=0.01204 *q*=0.02377<br>branch ω (mean)=0.314	Not allowed	-5061.77
**M8: β and ω**	*p*=0.07563 *q*=0.18279<br>f_0=0.97860<br>**ω_1=6.5 (f_1=0.0214)**<br>branch ω (mean)=0.426	241 P 0.969<br>**247 H 0.997**<br>575 W 0.991<br>**582 K 0.999**<br>583 S 0.979<br>584 L 0.978<br>**585 E 1.000**<br>587 I 0.993	-5038.30

*posterior probabilities >0.95 by Bayes Empirical Bayes (BEB) analysis
**ω=dN/dS
***Amino acid positions shown are for human GBP2.
