# Supplementary figures and images for: A Rapidly Evolving Polybasic Motif Modulates Bacterial Detection by Guanylate Binding Proteins
Source: mBio. 2020 May 19;11(3):e00340-20. doi: 10.1128/mBio.00340-20 (PMC7240152; doi:10.1128/mBio.00340-20)

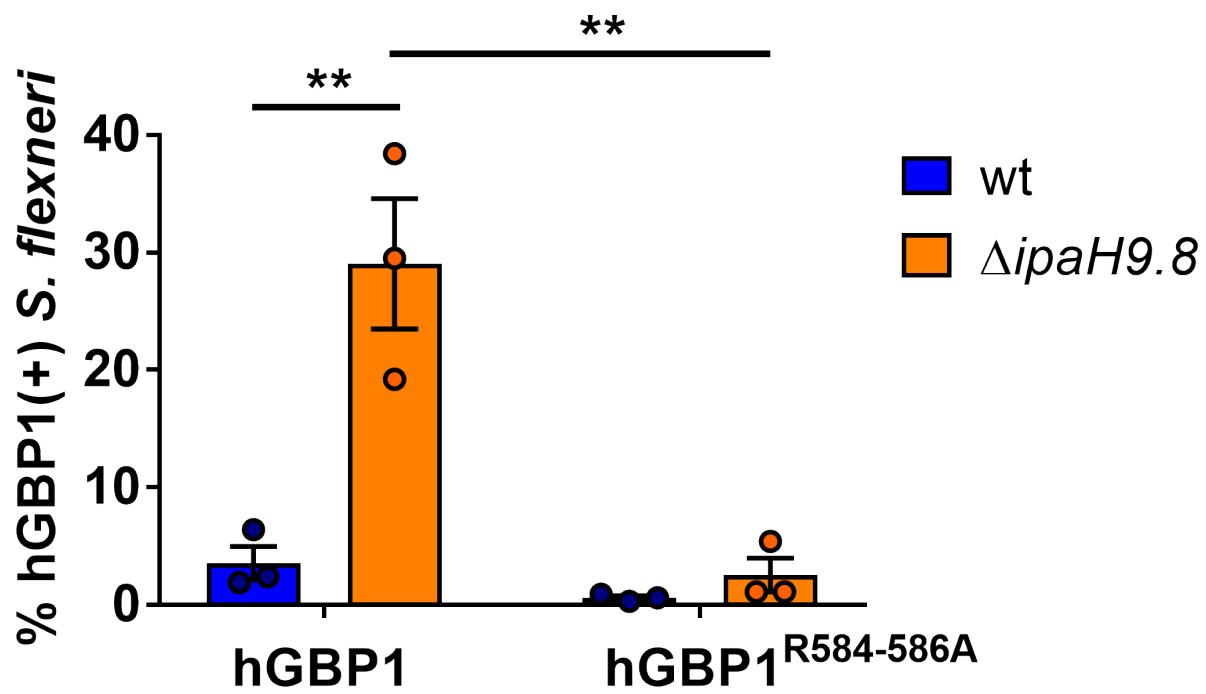

Supplement: FIG S3 [file mBio.00340-20-sf003.pdf]

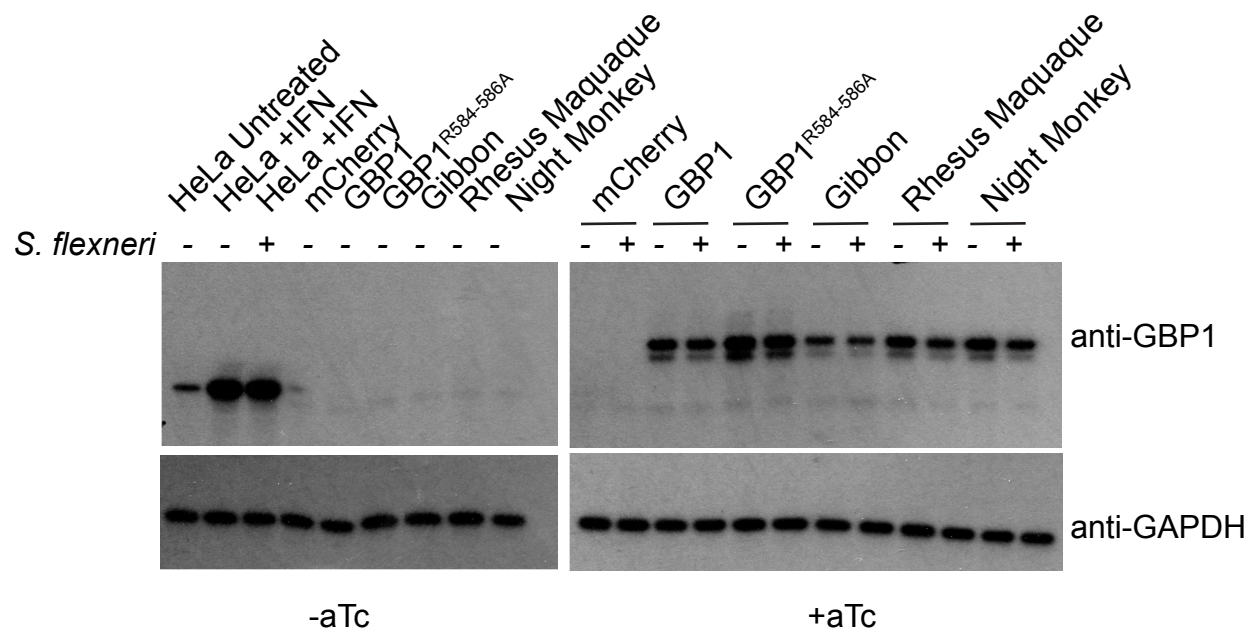

Supplement: FIG S4 [file mBio.00340-20-sf004.pdf]

A

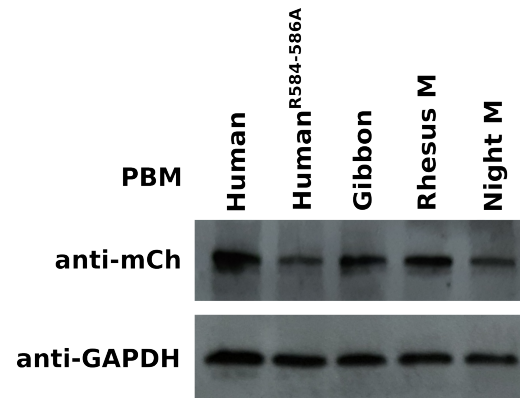

B

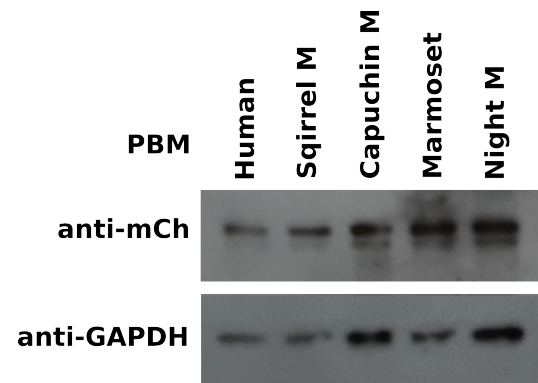

C

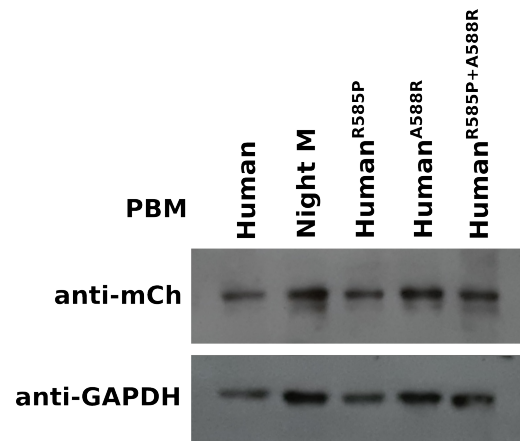

Supplement: FIG S5 [file mBio.00340-20-sf005.pdf]
