# Supplementary material for: A Rapidly Evolving Polybasic Motif Modulates Bacterial Detection by Guanylate Binding Proteins
Source: mBio. 2020 May 19;11(3):e00340-20. doi: 10.1128/mBio.00340-20 (PMC7240152; doi:10.1128/mBio.00340-20)
Supplement: TABLE S4 [file mBio.00340-20-st004.pdf]

Table S4A. Summary of positive selection in primate GBP2 (MEME, FEL, SLAC).

Model	Sites with evidence of positive selection (p-value)*
MEME	208 K (0.04)<br>209 G (0.04)<br>**247 H (0.03)**<br>296 L (0.01)<br>513 E (0.01)<br>565 N (0.02)<br>**582 K (0.01)**<br>**585 E (0.04)**<br>586 P (0.01)
FEL	**247 H (0.050)**<br>**582 K (0.033)**<br>**585 E (0.033)**
SLAC	No positively selected sites identified

Table S4B. Summary of positive selection in primate GBP2 using REL algorithm.

Sites with evidence of diversifying selection*	Posterior probability β>α
**H247**	**0.98**
**K582**	**0.98**
**E585**	**0.99**

Table S4C. Summary of positive selection in primate GBP2 using FUBAR algorithm.

Sites with evidence of diversifying selection*	Posterior probability β>α
M11	0.930
N161	0.925
E188	0.944
F234	0.926
A241	0.940
**H247**	**0.984**
E327	0.939
E334	0.924
G338	0.999
A549	0.909
**L550**	**0.936**
E564	0.905
**K582**	**0.989**
**E585**	**0.992**

*Amino acid positions shown are for human GBP2.
