# Supplementary material for: A Rapidly Evolving Polybasic Motif Modulates Bacterial Detection by Guanylate Binding Proteins
Source: mBio. 2020 May 19;11(3):e00340-20. doi: 10.1128/mBio.00340-20 (PMC7240152; doi:10.1128/mBio.00340-20)
Supplement: TABLE S5 [file mBio.00340-20-st005.pdf]

Table S5. Oligonucleotides used in this study.

Oligo Name	Sequence
pmCherry-hGBP1DBglII-F	5'-GCTGTACAAGTCCGGACTCAGGTCCATGGCATCAGAGATC-3'
pmCherry-hGBP1DBglII-R	5'-GATCTCTGATGCCATGGACCTGAGTCCGGACTTGTACAGC-3'
attB1-mCherry-F	5'-GGGGACAAGTTTGTACAAAAAAGCAGGCTGCCACCATGGTGAGCAAGGGCGAGG-3'
attB2-hGBP1DC_BglII-R	5'-GGGGACCACTTTGTACAAGAAAGCTGGGTCTTAGAGATCTTGTATCTCATTTTTCATT ATTCTGCTTTC-3'
Gibbon_PBM-F	5'-ATGAGATACAAGATCTCCAGGCGAAAATGCGTCCGCGCCGTCCGTGTACCATAAGCTAA GATCTCTAAGACCCA-3'
Gibbon_PBM-R	5'-TGGGTCTTAGAGATCTTAGCTTATGGTACACGGACGGCGCGGACGCATTTTCGCCTGGA GATCTTGTATCTCAT-3'
Rhesus Macaque_PBM-F	5'-ATGAGATACAAGATCTCCAGAAGAAAATGCGTCAGCGCCGTACCTGTACCATAAGCTAA GATCTCTAAGACCCA-3'
Rhesus Macaque_PBM-R	5'-TGGGTCTTAGAGATCTTAGCTTATGGTACAGGTACGGCGCTGACGCATTTTCTTCTGGA GATCTTGTATCTCAT-3'
Night Monkey_PBM-F	5'-ATGAGATACAAGATCTCCAGAACGCGATGCGTCCGCCGCGTCGCCGTTGTACCATAAG CTAAGATCTCTAAGACCCA-3'
Night Monkey_PBM-R	5'-TGGGTCTTAGAGATCTTAGCTTATGGTACAACGGCGACGCGGCGGACGCATCGCGTTC TGGAGATCTTGTATCTCAT-3'
Capuchin_PBM-F	5'-ATGAGATACAAGATCTCCAGAACGCCATGAAGCCCCCTCGGCGCAGATGTACCATAAG CTAAGATCTCTAAGACCCA-3'
Capuchin_PBM-R	5'-TGGGTCTTAGAGATCTTAGCTTATGGTACATCTGCGCCGAGGGGGCTTCATGGCGTTC TGGAGATCTTGTATCTCAT-3'
Squirrel Monkey_PBM-F	5'-ATGAGATACAAGATCTCCAGAACGCCATGAGACCCCGCAAGAGATGTACCATAAGCTAA GATCTCTAAGACCCA-3'
Squirrel Monkey_PBM-R	5'-TGGGTCTTAGAGATCTTAGCTTATGGTACATCTCTTGCGGGGTCTCATGGCGTTCTGGAG ATCTTGTATCTCAT-3'
Marmoset_PBM-F	5'-ATGAGATACAAGATCTCCAGAACGCCATGAAGAACGTGTTCTTCCCCCTGTCCCGGCGCT GTACCATAAGCTAAGATCTCTAAGACCCA-3'
Marmoset_PBM-R	5'-TGGGTCTTAGAGATCTTAGCTTATGGTACAGCGCCGGGACAGGGGGAAGAACACGTTCTT CATGGCGTTCTGGAGATCTTGTATCTCAT-3'
attB2-hGBP1-R	5'-GGGGACCACTTTGTACAAGAAAGCTGGGTCTTAGCTTATGGTACATGCCTTTCGTC-3'
hGBP1_R585P-F	5'-CAGACGAAAATGAGACCACGAAAGGCATGTACCATAAGC-3'
hGBP1_R585P-R	5'-GCTTATGGTACATGCCTTTCGTGGTCTCATTTTCGTCTG-3'
hGBP1_A588R-F	5'-CAGACGAAAATGAGACGACGAAAGCGATGTACCATAAGC-3
hGBP1_A588R-R	5'-GCTTATGGTACATCGCTTTCGTCGTCTCATTTTCGTCTG-3'
hGBP1_R585P_A588R-F	5'-CTCCAGACGAAAATGAGACCACGAAAGCGATGTACCATAAGC-3'
hGBP1_R585P_A588R-R	5'-GCTTATGGTACATCGCTTTCGTGGTCTCATTTTCGTCTGGAG-3'
Squirrel Monkey GBP2 C-Terminus gBlock	5'- CTTCTACAAACTGATCAGTCACTCTCAGAAAAGGAAAAAGCGCTTGAAGTGGAACGTGTAAAGGCTGAATCT GCCGAAGCTGCAAAGAAAATGTTGGAGGAAATACAAAAGAAGAACCAGCAGAT GATGGAACAGAAAGAGAAGAGTTATCAGGAACATGTGAAACAATTGACTGAGAAGATGGAGAG TGATAGGGCCCAATTAATAGCGGAGCAAGAGAAGACCATCGATGTTAAACTTAAGGAACAGGAACGCCTTC TCAAAGAGGGATTCGAGCTTGAGAGCAAGAGACTTCAAAAAGAGATACAAGATATCA AGAAAAGACGCAGATCATCATGTAACATACTCTAATGATCATAATCAGCCA-3'
